# Oncogenic Function of *DACT1* in Colon Cancer through the Regulation of β-catenin

**DOI:** 10.1371/journal.pone.0034004

**Published:** 2012-03-21

**Authors:** Guohong Yuan, Chongkai Wang, Chaolai Ma, Ning Chen, Qinghe Tian, Tonglin Zhang, Wei Fu

**Affiliations:** Department of General Surgery, Peking University Third Hospital, Beijing, China; Indiana University School of Medicine, United States of America

## Abstract

The Wnt/β-catenin signaling pathway plays important roles in the progression of colon cancer. *DACT1* has been identified as a modulator of Wnt signaling through its interaction with Dishevelled (Dvl), a central mediator of both the canonical and noncanonical Wnt pathways. However, the functions of *DACT1* in the WNT/β-catenin signaling pathway remain unclear. Here, we present evidence that *DACT1* is an important positive regulator in colon cancer through regulating the stability and sublocation of β-catenin. We have shown that *DACT1* promotes cancer cell proliferation *in vitro* and tumor growth *in vivo* and enhances the migratory and invasive potential of colon cancer cells. Furthermore, the higher expression of *DACT1* not only increases the nuclear and cytoplasmic fractions of β-catenin, but also increases its membrane-associated fraction. The overexpression of *DACT1* leads to the increased accumulation of nonphosphorylated β-catenin in the cytoplasm and particularly in the nuclei. We have demonstrated that *DACT1* interacts with GSK-3β and β-catenin. *DACT1* stabilizes β-catenin via *DACT1*-induced effects on GSK-3β and directly interacts with β-catenin proteins. The level of phosphorylated GSK-3β at Ser9 is significantly increased following the elevated expression of *DACT1*. *DACT1* mediates the subcellular localization of β-catenin via increasing the level of phosphorylated GSK-3β at Ser9 to inhibit the activity of GSK-3β. Taken together, our study identifies *DACT1* as an important positive regulator in colon cancer and suggests a potential strategy for the therapeutic control of the β-catenin-dependent pathway.

## Introduction

Mutations and the dysregulated expression of components of the ancient Wnt signaling pathway are linked to oncogenesis in multiple systems, and have been particularly implicated in the initiation of colon cancer [1], [2], [3], [4], [5], [6], [7], [8]. Over 90% of colorectal cancers originate from active mutations in the Wnt pathway [1]. Mutations have been described in the adenomatous polyposis coli (*APC*) gene in cases of familial adenomatous polyposis (FAP), and this mutation also occurs in a high proportion of sporadic colorectal cancers [9], [10]. *APC* mutations represent an early event in colorectal tumorigenesis [11].

β-catenin (official symbol CTNB1) is considered to be a central player in the Wnt signaling pathway. Although Wnt activation can occur through mutations that affect phosphorylation sites within exon 3 of β-catenin in a minority of colorectal tumors [12], [13], many other components of the Wnt signaling pathway contribute to colorectal cancer via dysregulating the activity or localization of β-catenin [14], [15].

The *DACT1* (Dapper1/Dpr1) gene, located at chromosome 14q22.3, encodes a 836 amino acid protein with a putative leucine zipper (LZ) domain in the amino-terminal end and a consensus PDZ binding (PDZ-B) motif in the carboxy-terminal end that allows the *DACT1* protein to interact with the Dishevelled (Dvl) PDZ domain [16]. Bioinformatic analyses have revealed that *DACT1* mRNA is expressed in the amnion, fetal brain, eye, heart, adult brain medulla, gastric cancer (signet ring cell features), RER+ colon tumor, acute lymphoblastic leukemia, germ cell tumor, chondrosarcoma, and parathyroid tumors [17]. Furthermore, based on the evolutionary and functional conservation of Wnt signaling molecules, as well as the human chromosomal localization of DACT1, the *DACT1* gene is also predicted to be a potent cancer-associated gene [17]. *DACT1* has been reported to be downregulated in hepatocellular carcinoma [18]. A recent report identified a correlation between *DACT1* expression in lung cancer and poor histological grade, large tumor size, extent of tumor invasion, and lymph node metastasis [19].

Although some studies have shown associations between *DACT1* expression and cancer, the function of *DACT1* in the WNT/β-catenin signaling pathway remains unclear. One possible mechanism is that Dpr stabilizes GSK-3β and axin in the APC complex, as shown by co-immunoprecipitation studies [16]. Another possibility is that Dpr competes with Fz for binding to the PDZ domain of Dvl, thereby blocking the signal transduction via Dvl, and hence inhibits the Dvl-mediated stabilization of β-catenin [20]. Yau et al. reported that human Dpr1 was downregulated in hepatocellular carcinoma, and this downregulation was correlated with the cytoplasmic accumulation of β-catenin [18]. However, in this report, we have found that *DACT1* is overexpressed in colon cancer, and it acts to enhance cellular proliferation, migration and invasion in colon cancer cell lines. We have shown that *DACT1* interacts with GSK-3β and β-catenin. We have further demonstrated that *DACT1* stabilizes β-catenin via *DACT1*-induced effects on GSK-3β and directly interacts with β-catenin. We have also demonstrated *DACT1* inhibits the activity of GSK-3β via increasing the level of phosphorylated GSK-3β at Ser9, which alters the subcellular location of β-catenin. It particularly promotes β-catenin levels at the plasma membrane and in the nucleus.

## Results

### *DACT1* is overexpressed in human colon carcinoma

**Figure 1:**
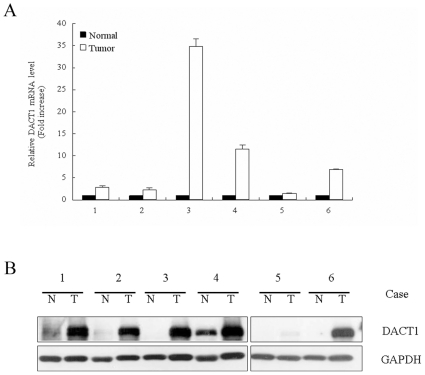
*DACT1* is highly expressed in human colon cancer cells. (A) *DACT1* mRNA levels were assayed using quantitative real-time RT-PCR analysis. Samples of colon adenocarcinoma (T, white bars) and normal-appearing control mucosa (N, black bars) were analyzed in six cases of colon cancer. (B) Expression of *DACT1* protein in human colon adenocarcinoma (T) and normal-appearing control mucosa (N) in six cases, as determined by Western immunoblotting analysis. The expression of GAPDH was used as the loading control.

To identify the potential roles of *DACT1* in the development and progression of colonic carcinoma, we used quantitative real-time PCR (qRT-PCR) to assess the level of gene expression. We compared the expression in six cancer tissues to those in six paired samples of normal control colonic mucosa. The results showed that the level of *DACT1* mRNA was significantly elevated in all six samples of colon cancer (Figure 1A). The gene expression data were further confirmed by Western blotting, which were performed with the use of soluble cell lysates prepared from surgical colectomy specimens. The results confirmed that high levels of *DACT1* protein are present in colon cancer (Figure 1B).

### *DACT1* enhances cellular proliferation *in vitro* and colon tumorigenesis *in vivo*

**Figure 2:**
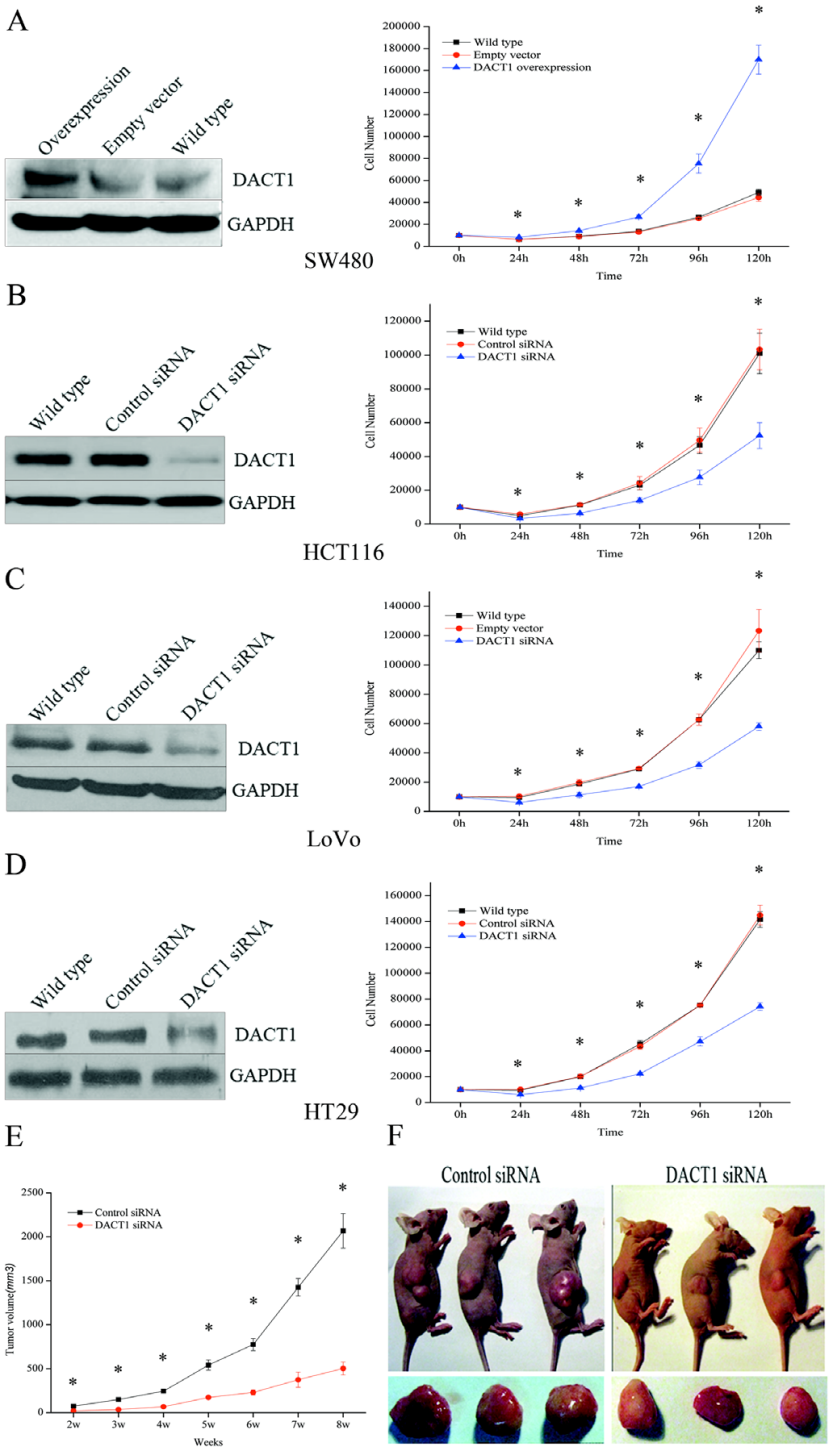
*DACT1* enhances cellular proliferation and colon tumorigenesis. (A) Left: overexpression of *DACT1* in stably transfected SW480 cells. Right: growth curve proliferation assay in *DACT1*-transfected SW480 cells (mean ± SEM; n = 3, *p<0.05 versus empty vector-transfected cells). (B, C, D) Left: *DACT1* expression in wild-type, control siRNA-transfected, and *DACT1* siRNA-transfected HCT116, LoVo and HT29 cells. Right: growth curve assays in *DACT1* siRNA-transfected HCT116, LoVo and HT29 cells (mean ± SEM; n = 3, *p<0.05 versus wild-type cells). (E) Average tumor volume assessed weekly after the injection of animals with control siRNA-or *DACT1* siRNA-transfected HCT116 cells (mean ± SEM; n = 3, *p<0.05). (F) Representative pictures eight weeks after injection of mouse colon cancer cells with control siRNA or *DACT1* siRNA-transfected HCT116 cells.

To understand the function of *DACT1* in colon cancer, we explored the effect of ectopic *DACT1* expression on cellular growth in vitro in cell lines with different levels of endogenous *DACT1* expression. Following transfection with a *DACT1* cDNA expression construct, SW480 colon cancer cells, which express very low endogenous levels of *DACT1*, demonstrated a significant increase in cellular proliferation (Figure 2A). Conversely, cellular proliferation decreased when endogenous *DACT1* expression was silenced by stable transfection with siRNA vectors that targeted *DACT1* in the HCT116, LoVo and HT29 colon cancer cell lines, which have much higher endogenous levels of *DACT1* (Figure 2B–D). To confirm these results, endogenous *DACT1* expression was silenced by stable transfection with another siRNA vectors (*DACT1* siRNA1) that targeted distinct regions of *DACT1* in the HCT116 cells and the same results were observed (Figure S1A–B).

To examine the effects of the silencing of *DACT1* on colon tumor growth in vivo, HCT116 cells that stably expressed a control siRNA or *DACT1* siRNA were used. Cells (5×10^6^) were injected into the subcutaneous tissue of the nude mouse (n = 8 animals per group). Tumors were measured weekly with a vernier caliper, and their volumes were calculated according to the following formula: π/6×length×width^2^ [21]. All of the animals injected with HCT116 cells that expressed control siRNA developed tumors by 14 days after injection, while only 75% (6/8) of the *DACT1* siRNA animals developed tumors. Fifty-six days after injection, the tumors of the control siRNA animals were significantly larger (Figure 2F). The mean tumor volume was 2,068.78±561.57 mm^3^ in mice injected with HCT116 cells that expressed control siRNA, compared to the mean tumor volume in mice injected with HCT116 cells that expressed *DACT1* siRNA (503.46±178.90 mm^3^) (Figure 2E). These data demonstrate the important role of *DACT1* in promoting the growth of colon cancer cells.

### *DACT1* enhances the migration and lack of anchorage of colon cancer cells

**Figure 3:**
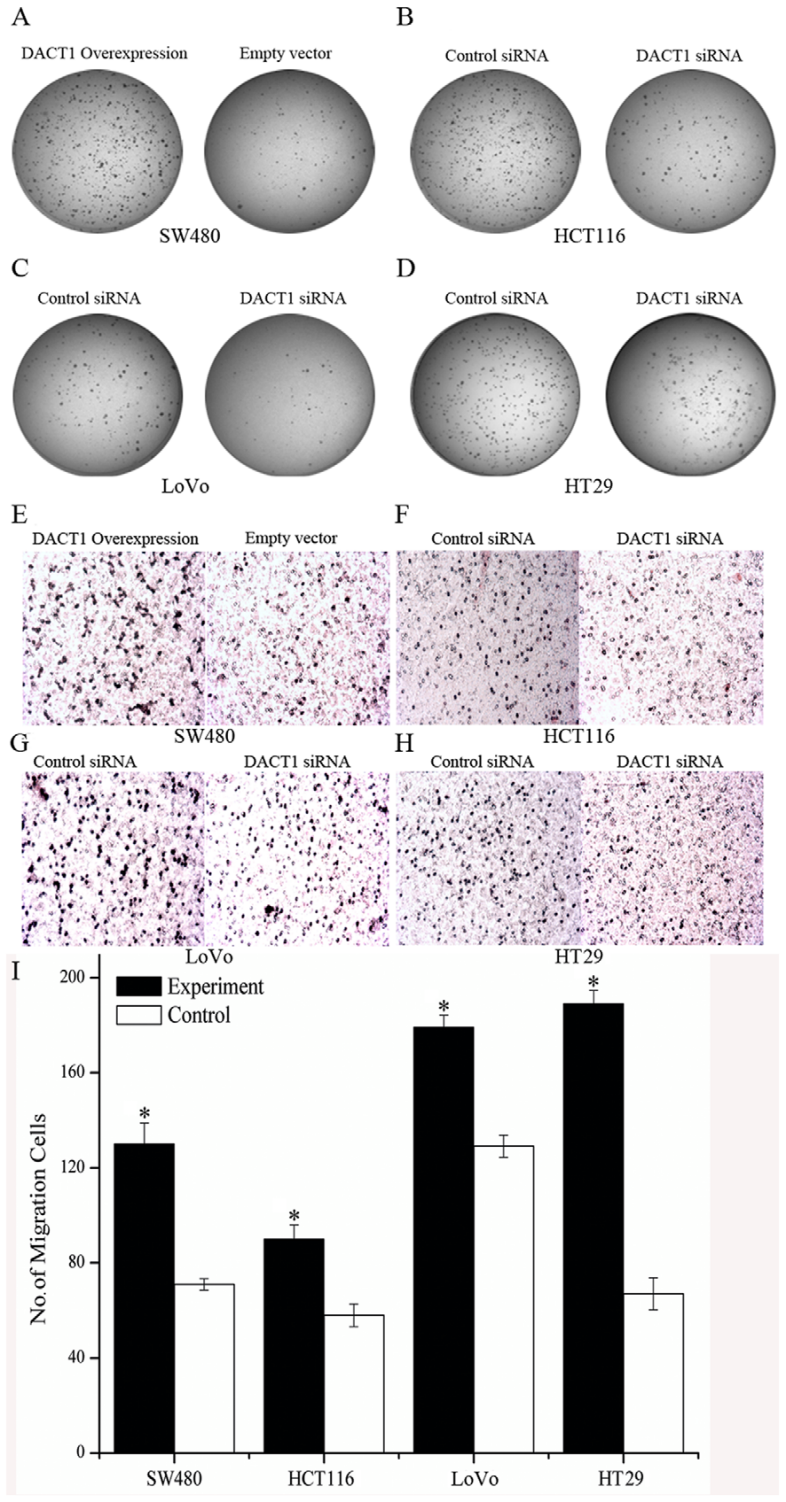
*DACT1* enhances anchorage independence and the migratory potential of colon cancer cells. (A) Soft agar assay in *DACT1*-transfected SW480 cells after 14 days of incubation. (B, C, D) Soft agar assay in siRNA-transfected HCT116, LoVo and HT29 cells after 14 days of incubation. (E) Representative images are shown for the overexpression of *DACT1* in stably transfected SW480 cells. Cells were seeded in a transwell chamber and allowed to migrate across the chamber toward cell-specific conditioned medium for 24 h. Photomicrographs of stained migrating cells were taken under brightfield illumination (20×). (F, G, H) Representative images are shown for *DACT1* siRNA-transfected HCT116, LoVo and HT29 cells. Cells were seeded in a transwell chamber and allowed to migrate across the chamber toward cell-specific conditioned medium for 24 h. Photomicrographs of stained migrating cells were taken under brightfield illumination (20×). (I) Quantification of migration assay. Results were obtained from three separate experiments each performed in triplicate. Migration was determined by counting cells in six random microscopic fields per well (mean ± SEM; *p<0.05 versus control group cells).

Anchorage-independent proliferation is a hallmark of oncogenic transformation and is regarded to be conducive to the proliferation of cancer cells away from their original site. With this knowledge, we examined the capacity of *DACT1* to drive the anchorage-independent growth of colon cancer cells by soft agar colony formation assays. The overexpression of *DACT1* enhanced the anchorage-independent proliferation of SW480 cells (Figure 3A). Downregulation of *DACT1* reduced the anchorage-independent potential of HCT116, LoVo and HT29 cells (Figure 3B–D and S1C). Furthermore, we observed that *DACT1* overexpression enhanced the migratory potential of SW480 cells by transwell assays (Figure 3E and I). Conversely, the migratory potential of cells was decreased when endogenous *DACT1* expression was silenced by stable transfection with siRNA vectors that targeted *DACT1* in HCT116, LoVo and HT29 cells (Figure 3F–I). Following these results, we conclude that *DACT1* enhances anchorage independence in colon cancer cells and their migratory potential.

### *DACT1* enhances invasion of colon cancer cells *in vitro* or *in vivo*

**Figure 4:**
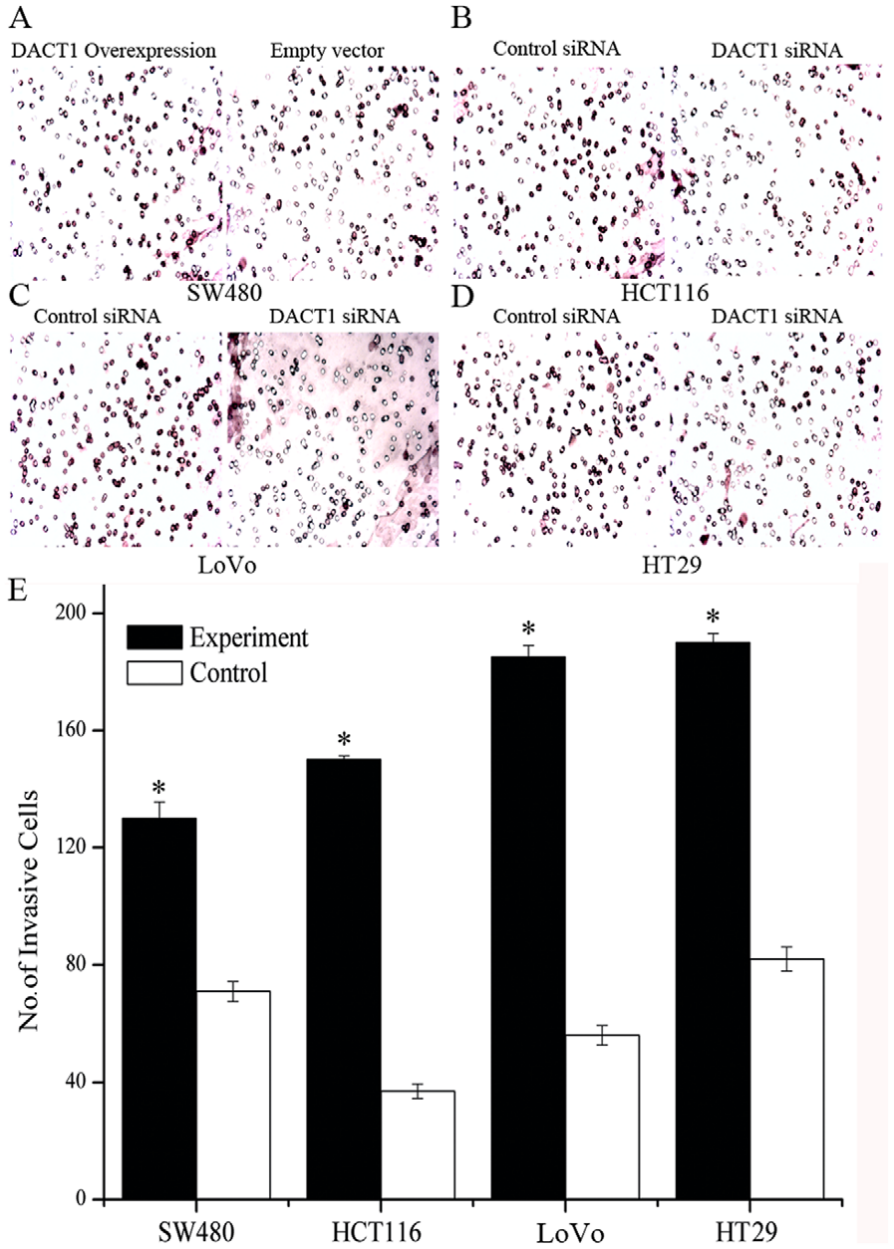
*DACT1* enhances invasion of colon cancer cells in vitro or in vivo. (A) Representative images are shown for the overexpression of *DACT1*-transfected SW480 cells. Cells were seeded in an invasive chamber and allowed to invade the chamber toward cell-specific conditioned medium for 24 h. Photomicrographs of stained invading cells were taken under brightfield illumination (20×). (B, C, D) Representative images are shown for *DACT1* siRNA-transfected HCT116, LoVo and HT29 cells. Cells were seeded in an invasion chamber and allowed to invade the chamber toward cell-specific conditioned medium for 24 h. Photomicrographs of the stained invading cells were taken under brightfield illumination (20×). (E) Quantification of the migration assay. Results were obtained from three separate experiments each performed in triplicate. Invasion was determined by counting cells in six random microscopic fields per well (mean ± SEM; *p<0.05 versus control group cells).

The most dangerous feature of malignancy is the invasive and metastatic potential of tumor cells. To test whether cell invasion was affected by *DACT1*, the effects of *DACT1* on cell invasion through Matrigel was investigated. Overexpression of *DACT1* enhanced the invasion of SW480 cells (Figure 4A and E). Downregulation of *DACT1* reduced the invasive potential of HCT116, LoVo and HT29 cells through the Matrigel (Figure 4B–E). To examine the effects of *DACT1* silencing on the invasion of colon cancer cells in vivo, we infected HCT116 cells that expressed a control siRNA or *DACT1* siRNA. Then, 5×10^6^ cells were injected into the subcutaneous tissue of each nude mouse (n = 8 animals per group). Fifty-six days later, the tumors of six out of eight control siRNA-transfected nude mice had invaded into the musculature, while only two of the tumors in *DACT1* siRNA-transfected animals had invaded into the musculature. These data further show that *DACT1* did affect the invasive potential of colon cancer cells in vitro and in vivo.

### *DACT1* regulates the cell cycle though increasing β-catenin levels

**Figure 5:**
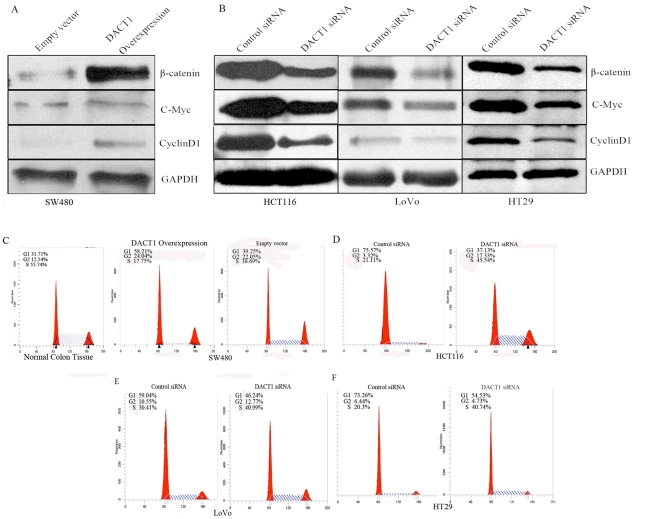
*DACT1* regulates the cell cycle through increasing levels of β-catenin. (A) Representative Western blots of empty vector-transfected and *DACT1*-transfected SW480 cells. Overexpression of *DACT1* results in the upregulation of β-catenin and the T cell factor (TCF) target genes, cyclin D1 and c-Myc. GAPDH was used as the loading control. (B) Representative Western blots of HCT116 and LoVo and HT29 cells expressing control siRNA or *DACT1* siRNA. Silencing of *DACT1* expression in HCT116 and LoVo and HT29 cells decreased levels of total β-catenin, cyclin D1 and c-Myc. GAPDH was used as the loading control. (C) The effect of *DACT1* overexpression on the cell cycle profile of SW480 cells. Three days after serum starvation, cells were analyzed for their cell cycle profile by FACS. The percentage of cells in the G1, G2 and S phases are shown. (D, E, F) The effect of *DACT1* siRNA transfection on the cell cycle profile of HCT116, LoVo and HT29 cells. Three days after serum starvation, cells were analyzed for their cell cycle profile by FACS. The percentage of cells in the G1, G2 and S phases are shown.

To elucidate the mechanism which caused the above results further, we tested the protein levels of the β-catenin and the Wnt/β-catenin target genes, cyclin D1 [8] and c-Myc [22]. Our experiment showed that overexpression of *DACT1* resulted in a significant increase in the total levels of β-catenin, cyclin D1 and c-Myc in SW480 cells (Figure 5A). Conversely, *DACT1* siRNA, which mediated the silencing of endogenous *DACT1* expression, decreased the total levels of β-catenin, cyclin D1 and c-Myc levels in HCT116, LoVo and HT29 cells (Figure 5B).

Considering that cyclin D1 and c-Myc are related to cell cycle regulation, we examined the effect of *DACT1* overexpression and silencing on cell cycle regulation in colon cancer cells. In these experiments, all of the cells were synchronized at the G0/G1 phases by serum starvation. Cell cycle profiling by FACS indicated that the overexpression of *DACT1* in SW480 cells was associated with an increase in the proportion of cells in the G0/G1 phases (Figure 5C). Consistently, silencing of *DACT1* expression with siRNA resulted in an increased number of cells in the S+G2/M phases in the HCT116, LoVo and HT29 cell lines (Figure 5D–F). These results suggest that the S+G2/M accumulation of cells after *DACT1* silencing is specifically associated with the loss of *DACT1* protein. Collectively, these results indicate that *DACT1* promotes the proliferation of colon cancer cells by facilitating the transition from the G1 to the S phase of the cell cycle.

Besides cyclin D1, CDK4 is another key factor that facilitates the G1/S transition. We noted that the overexpression of *DACT1* in SW480 cells increased the expression of CDK4, while CDK4 levels were significantly reduced in HCT116, LoVo and HT29 cells that expressed *DACT1* siRNA (Figure S2). Together, these results clearly show that *DACT1* stimulates cell proliferation and tumor growth by promoting the entry of cells into the cell cycle through increasing levels of β-catenin.

### *DACT 1* enhances migratory and invasive potential of colon cancer cells through changing the subcellular location of β-catenin

**Figure 6:**
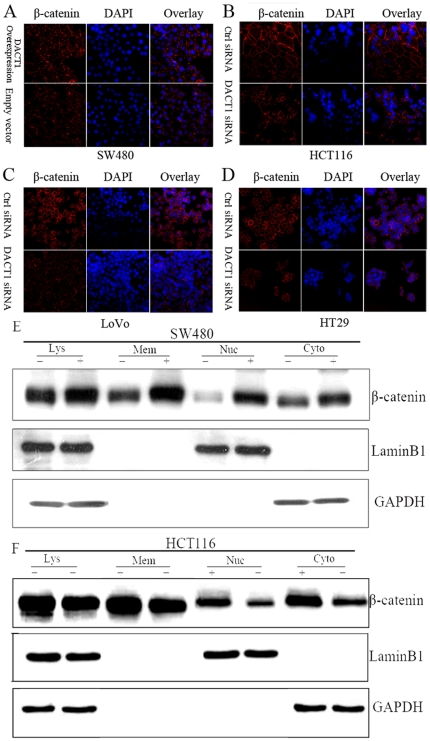
*DACT1* enhances the migratory and invasive potential of colon cancer cells through changing the subcellular location of β-catenin. (A) Photomicrographs of empty vector and *DACT1*-overexpressing SW480 cells immunostained with an anti-β-catenin antibody (red). (B, C, D) Photomicrographs of control siRNA and *DACT1* siRNA in HCT116, LoVo and HT29 cells immunostained with an anti-β-catenin antibody (red). (E) Representative blots of β-catenin levels in membrane (Mem), nuclear (Nuc), and cytoplasmic (Cyto) fractions and total lysates (Lys) in SW480 cells. Laminin B (nuclear expression) and GAPDH (cytoplasmic expression) were used as the loading controls. “+” represents the overexpression *DACT1*. “−” is empty vector. (F) Representative blots of β-catenin levels in membrane (Mem), nuclear (Nuc), and cytoplasmic (Cyto) fractions and total lysates (Lys) in HCT116 cells. Laminin B (nuclear expression) and GAPDH (cytoplasmic expression) were used as the loading controls. “+” represents control siRNA. “−” is *DACT1* siRNA.

To reveal the means by which *DACT1* increases β-catenin/TCF-regulated gene expression, the precise location of β-catenin was observed under a laser scanning confocal microscope (LSCM) by immunofluorescence. The results showed that the higher expression of *DACT1* not only increased the nuclear and cytoplasmic fractions of β-catenin, but also increased its membrane-associated fraction. The change in the level of the β-catenin membrane-associated fraction was the most significant finding (Figure 6A–D and S1D).

To confirm these interesting results, extracts from control/*DACT1*-overexpressing SW480 cells and control/*DACT1*-silenced HCT116 cells were separated into membrane, cytoplasmic, and nuclear fractions. Then, the relative abundance of β-catenin in these fractions was analyzed by Western blotting (Figure 6E and F). Previous reports have demonstrated an important role for β-catenin in cell adhesion as a part of a protein complex that includes E-cadherin [23]. The E-cadherin expression pattern in colon cancer cells was unaltered by *DACT1* silencing or overexpression (Figure S3). These data suggest that increased levels of *DACT1* affect β-catenin levels and β-catenin/TCF-dependent transcription by the activation of the Wnt signaling pathway. They also suggest the possibility that *DACT1* enhances the migratory and invasive potential of colon cancer cells via β-catenin-mediated stabilization of the adherens junction.

### Correlation between *DACT1* and membrane-associated β-catenin expression *in vitro* and *in vivo*

**Figure 7:**
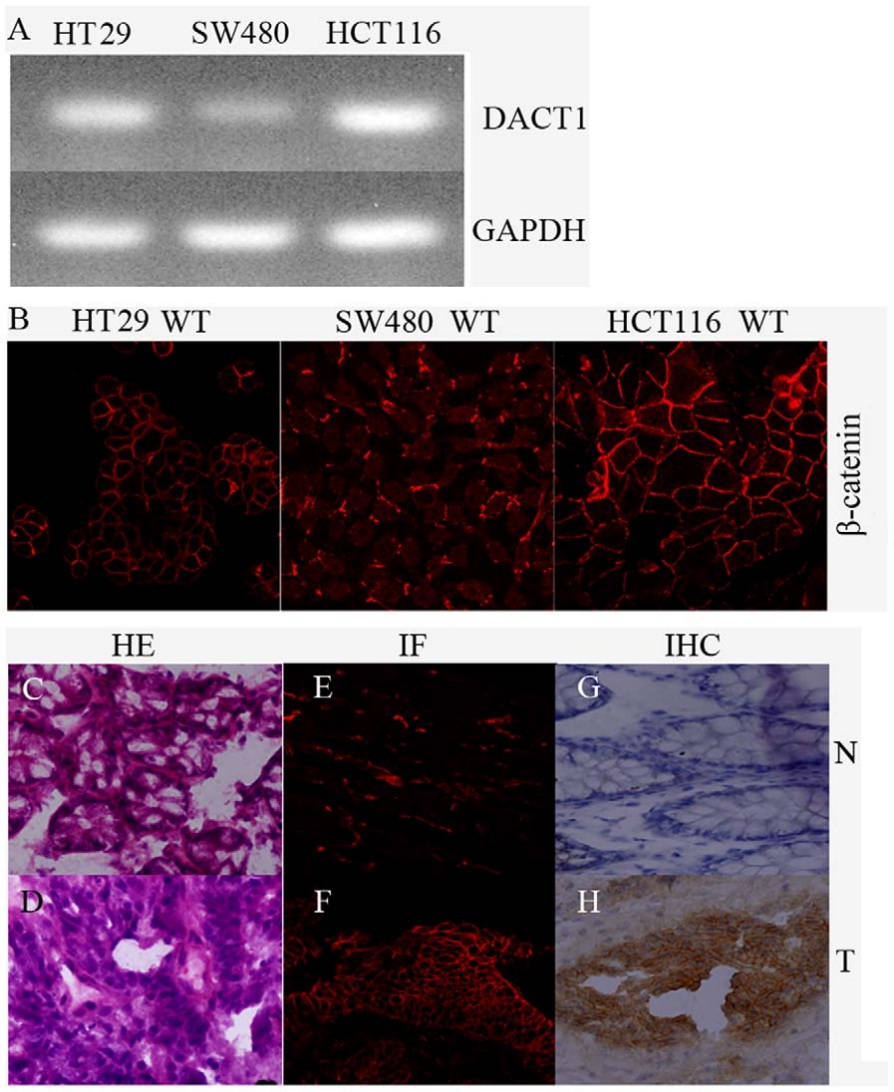
Correlation between *DACT1* levels and membrane-associated β-catenin expression in colon cell lines and colon cancer tissue. (A) Semiquantitative RT-PCR analysis of *DACT1* expression in HCT116, HT29 and SW480 cells. GAPDH was used as the control. (B) Immunofluorescence analysis of *DACT1* and β-catenin expression in HCT116, HT29 and SW480 cells. Original magnification, 40×. (C, D) HE staining of samples of normal human colon mucosa (N) and adenocarcinoma (T) tissues by an anti-β-catenin antibody. Original magnification, 40×. (E, F) immunofluorescence (IF) staining of samples of normal human colon mucosa (N) and adenocarcinoma (T) tissues by an anti-β-catenin antibody. Original magnification, 40×. (G, H) Immunohistochemical (IHC) staining of samples of normal human colon mucosa (N) and adenocarcinoma (T) tissues by an anti-β-catenin antibody. Original magnification, 40×.

From the results of the above experiments, we found that *DACT1* was highly expressed in human colon cancers and that it promoted the cell growth, migration and invasion potential of colon cancer cells through its effects on β-catenin signaling. Based on these findings, we hypothesized that *DACT1* and the expression levels of membrane-associated β-catenin would be positively correlated in colon cancer cell lines and primary colon cancers. As shown in Figure 7A and B, the levels of *DACT1* and β-catenin in colon cancer cell lines were correlated. The highest levels of β-catenin at the cell membrane were observed in HCT116 cells with high levels of endogenous *DACT1*. Intermediate levels of β-catenin at the cell membrane and *DACT1* proteins were found in HT29 cells. Both membrane-associated β-catenin and *DACT1* were minimally expressed in SW480 cells.

To study the relationship between *DACT1* and membrane-associated β-catenin expression in colon cancer further, immunohistochemical and immunofluorescence analyses were performed of β-catenin in human colon normal and adenocarcinoma tissues (the six above-mentioned cases). We observed focal staining of membrane-associated β-catenin in all six tumors (100%; Figure 7C–H). These results correlate well with the previous results with regards to β-catenin expression in colon cancer cell lines. These results confirm that elevated levels of membrane-associated β-catenin may be dependent on the elevated expression of *DACT1*.

### *DACT1* interacts with β-catenin and the components of the β-catenin destruction complex to stabilize β-catenin

**Figure 8:**
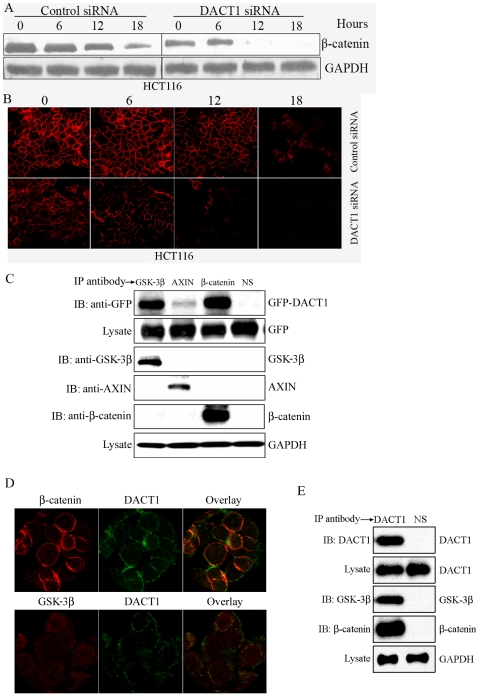
*DACT1* interacts with β-catenin and components of the β-Catenin destruction complex to stabilize β-catenin. (A) Western blot assays in HCT116 cells were performed to determine β-catenin stability. (B) Immunofluorescence assays in HCT116 cells were performed to determine β-catenin stability. (C) Cell lysates from SW480 cells transfected with GFP-*DACT1* were subjected to immunoprecipitation (IP) with anti-axin, anti-GSK-3β, or anti-β-catenin antibodies. Immunocomplexes were resolved by SDS-PAGE and subjected to Western blot analyses with an anti-GFP antibody. Blotting with an anti-GAPDH antibody showed equal loading. (D) Subcellular co-localization of endogenous *DACT1* and β-catenin, *DACT1* and GSk-3β in HT29 cells. (E) Cell lysates from HT29 cells were subjected to immunoprecipitation (IP) with an anti-*DACT1* antibody. Immunocomplexes were resolved by SDS-PAGE and subjected to Western blot analyses with anti-GSK-3β, or anti-β-catenin antibodies. Blotting with an anti-GAPDH antibody showed equal loading.

We were interested in clarifying the mechanism by which elevated levels of *DACT1* resulted in increased β-catenin expression. In the absence of Wnt ligands, cytoplasmic levels of β-catenin are regulated by the destruction complex that contains axin, APC, and glycogen synthase kinase-3β (GSK-3β), where β-catenin is phosphorylated by GSK-3β at multiple serine and threonine residues in its N terminus. Phosphorylated β-catenin is then ubiquitinated, leading to its rapid proteasomal degradation [4], [24], [25], [26], [27], [28]. To determine whether *DACT1* increases β-catenin levels by influencing β-catenin stability, HCT116 cells (with or without *DACT1* silencing) were incubated with 10 µg/ml cycloheximide (CHX) to prevent new β-catenin synthesis. Then, β-catenin levels were measured by Western blotting to reflect the rate of β-catenin protein degradation. Silencing of *DACT1* expression significantly increased the β-catenin degradation rate, resulting in an evident reduction in the levels of remaining β-catenin (Figure 8A). This effect of *DACT1* on β-catenin stability was confirmed by an immunofluorescence assay. We found that β-catenin was degraded more rapidly in HCT116 cells that expressed *DACT1* siRNA than in HCT116 cells expressing control *DACT1* siRNA (Figure 8B).

To ascertain the mechanism by which *DACT1* influences β-catenin stability, we examined whether or not *DACT1* interacts with the components of the multiprotein complex that regulates β-catenin stability. Our co-immunoprecipitation analysis revealed colocalization of *DACT1* and GSK-3β, *DACT1* and axin in SW480 cells stably transfected with a GFP-tagged *DACT1* construct (Figure 8C). Moreover, *DACT1* did interact with β-catenin in SW480 cells that overexpressed *DACT1* (Figure 8C). We then examined the colocalization of *DACT1* with GSK-3β, and *DACT1* with β-catenin in wild type HT29 cells (without APC or β-catenin mutations) (Figure 8D–E). These consistent results indicated that *DACT1* interferes with the GSK-3β-dependent phosphorylation of β-catenin by the destruction complex, and *DACT1* affects β-catenin stability by interacting with β-catenin.

### *DACT1* affects the subcellular localized β-catenin through inhibiting GSK-3β activity

**Figure 9:**
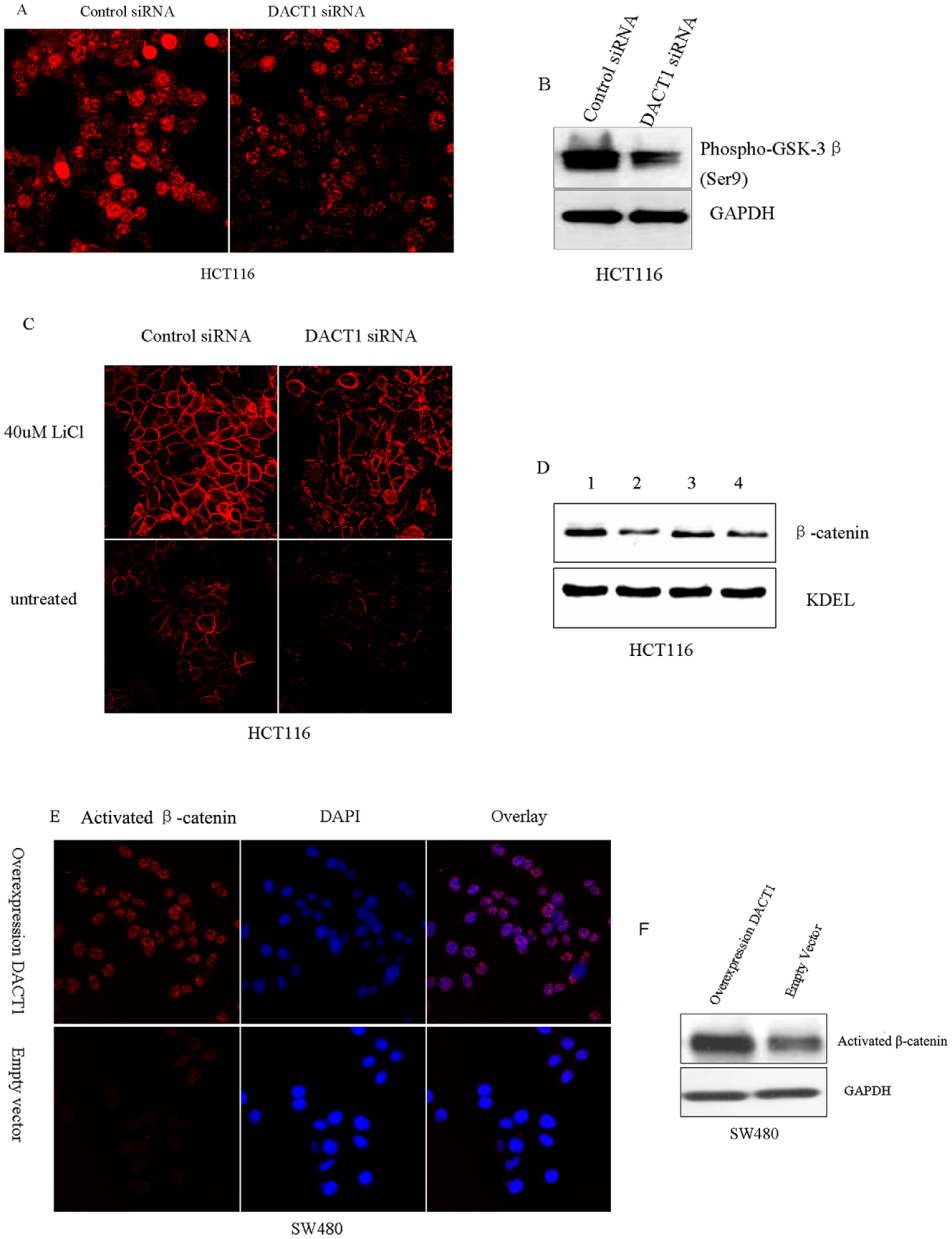
*DACT1* affects the subcellular localization of β-catenin through interacting with GSK-3β. (A) Photomicrographs of control siRNA and *DACT1* siRNA expressing HCT116 cells immunostained with an anti-phospho-GSK-3β(Ser9) antibody (red). Silencing of *DACT1* in HCT116 cells decreases levels of phospho-GSK-3β(Ser9). (B) Representative Western blots of HCT116 cells expressing control siRNA and *DACT1* siRNA. Silencing of *DACT1* in HCT116 cells decreases levels of phospho-GSK-3β(Ser9). GAPDH was used as the loading control. (C) HCT116 cells expressing control siRNA and *DACT1* siRNA were treated for 1 h with GSK-3β inhibitory drugs (40 mM LiCl). Cells were stained and analyzed by microscopy to detect the expression of β-catenin. LiCl treatment of HCT116 cells increases the levels of β-catenin at the plasma membrane. (D) Representative Western blots of LiCl-treated HCT116 cells, which express either control siRNA or *DACT1* siRNA. LiCl treatment of HCT116 cells increases levels of β-catenin at the plasma membrane. KDEL was used as a loading control. 1: HCT116 cells expressing control siRNA that had been treated with 40 mM LiCl for 1 h. 2: HCT116 cells expressing control siRNA that had not been treated with LiCl. 3: HCT116 cells expressing *DACT1* siRNA that were treated by 40 mM LiCl for 1 h; 4: HCT116 cells expressing *DACT1* siRNA that had not been treated with LiCl. (E) Photomicrographs of control siRNA and *DACT1* siRNA expressing HCT116 cells immunostained with an anti-phospho-GSK-3β(Ser9) antibody (red). Silencing of *DACT1* in HCT116 cells decreases levels of phospho-GSK-3β(Ser9). (F) Representative Western blots of HCT116 cells expressing control siRNA and *DACT1* siRNA. Silencing of *DACT1* in HCT116 cells decreases levels of phospho-GSK-3β(Ser9). GAPDH was used as a loading control.

β-catenin is known to accumulate in the nucleus [29] and its levels are speculated to increase at the membrane lamellipodia, membrane adherens junctions and membrane ruffles [30], [31] in response to Wnt signaling or the drug-mediated inhibition of GSK-3β activity. Moreover, based on our findings, we speculated that *DACT1* inhibits GSK-3β. Some factors that inhibit GSK-3β via the phosphorylation of Ser9 have been reported [32], [33]. We observed the protein expression levels of GSK-3β with phosphorylated Ser9 in HCT116 cells (with or without *DACT1* silencing). The results showed that the levels of phosphorylated GSK-3β at Ser9 were significantly increased in HCT116 cells that expressed control siRNA (Figure 9A and B). To confirm that *DACT1* regulated the localization pattern of β-catenin through inhibiting GSK-3β, we tested the effect of GSK-3β inhibition on HCT116 cells that expressed either the control siRNA or the *DACT1* siRNA. We observed a clear and significant enhancement in cells that displayed membrane-associated β-catenin staining after they were treated with 40 mM LiC1 for 1 h (Figure 9C and D). We further tested the expression of activated β-catenin in SW480 cells. The results revealed the increased accumulation of nonphosphorylated β-catenin in cytoplasm and particularly in the nuclei of SW480 cells that overexpressed *DACT1* (Figure 9E and F). All of these results support the conclusion that *DACT1* affects the subcellular localization of β-catenin through the inhibition of GSK-3β activity.

## Discussion

### Increased expression of *DACT1* has oncogenic functions in colon cancer

Our studies have shown that *DACT1* is overexpressed in human colonic adenocarcinomas. This result is consistent with previous observation that *DACT1* was upregulated in invasive breast tumors [34]. Our studies have also revealed that β-catenin and Dvl-2 are upregulated along with *DACT1*, and demonstrate that there is a trend toward poor survival in patients with tumors that have higher scores for *DACT1* expression in colon adenocarcinomas [35]. These results suggest that the higher expression of *DACT1* may have oncogenic functions in colon cancer. However, *DACT1* has also been reported to be downregulated in hepatocellular carcinoma [18] and lung cancer [19], which suggests that the function of *DACT1* may be dependent on cellular context. Here, our observations show that the expression of *DACT1* enhances cellular proliferation and promotes tumor growth and metastasis in the SW480, HCT116, LoVo and HT29 cell lines with or without APC or β-catenin mutations.

### *DACT1* upregulates β-catenin levels in colon cancer cells

Xenopus Dpr, the ortholog of *DACT1*, was found to be a negative regulator in both Dvl-mediated canonical and noncanonical Wnt pathway by Cheyette et al. in 2002 [16]. Concurrently, Gloy et al. demonstrated that Fordo, another ortholog of *DACT1* promotes Wnt signaling in axis formation and in eye and neural development in Xenopus embryos [36]. To date, the function of DACT proteins in the regulation of signaling pathways remains ambiguous, but it is very clear that *DACT1* not only regulates embryonic development but may have an important role in tumorigenesis. Indeed, one study demonstrated that *DACT1* was downregulated in hepatocarcinoma, and this downregulation was associated with the methylation of its promoter-associated CpG island and significantly correlated with the cytoplasmic accumulation of β-catenin [18]. However, Jiang et al. reported that the expression of *DACT1* did not differ significantly between human colorectal tumors and control tissues, and the *DACT1* promoter was partially methylated [37]. *DACT1* expression in lung cancer was downregulated [19].

We found that *DACT1* upregulated β-catenin in colon cancer cell lines. The higher expression of *DACT1* not only increased the nuclear and cytoplasmic fractions of β-catenin, but also increased its membrane-associated fraction. In mammals, β-catenin is originally identified as a component associated with cadherins, which are Ca2+-dependent cell-cell adhesion molecules [38]. Besides its function in the cadherin complex, β-catenin has an essential role in the Wnt signaling pathway [39], [40], [41]. Our results showed that the E-cadherin expression pattern in colon cancer cells was unaltered by *DACT1* siRNA or *DACT1* overexpression. These results suggest that *DACT1* affects the level of β-catenin through the Wnt signaling pathway. Cyclin D1 and c-Myc are Wnt target genes. They can regulate the cell cycle and promote cell proliferation. We found that increased level of *DACT1* not only led to higher levels of total β-catenin and activated β-catenin, but increased the expression of the β-catenin/TCF target genes, cyclin D1 and c-Myc. Further studies showed that *DACT1* promoted the proliferation of colon cancer cells by facilitating their transition from the G1 to the S phase of the cell cycle. The overexpression of *DACT1* in the SW480 cell line resulted in an increase in the population of cells in the G0/G1 phases. While the expression of *DACT1* in cells with a *DACT1* knockdown resulted in an increase in the population of cells in the S+G2/M phases. Those results are consistent with the results that *DACT1* enhances cellular proliferation in vitro and colon tumorigenesis in vivo.

### The mechanisms of *DACT1*-induced upregulation of β-catenin in colon cancer cells

The stability of β-catenin is known to be properly regulated by the multiprotein complex containing axin, GSK-3β, and APC. In the absence of an activating Wnt signal, mediated via Wnt binding to the Frizzled/LRP5/6 coreceptor complex, cytoplasmic β-catenin is destabilized by the multiprotein complex. Axin acts as the scaffold of this complex and interacts with the other components, β-catenin, APC, and GSK-3β. The interaction of GSK-3β with axin in the complex facilitates efficient phosphorylation of β-catenin by GSK-3β. Phosphorylated β-catenin is then ubiquitinated, leading to its rapid proteasomal degradation. We found that *DACT1* binds to axin and GSK-3β in colon cancer cells. The results support the fact that *DACT1* interacts with the destruction complex to prevent the phosphorylation and subsequent ubiquitination of β-catenin. We also found that *DACT1* interacted with β-catenin in the plasma membrane, which indicated that *DACT1* may directly regulate the degradation of β-catenin, leading to the accumulation of cytoplasmic β-catenin. However, the mechanisms by which *DACT1* regulates this multiprotein complex and β-catenin are unclear.

*DACT1* has been identified as an interacting protein for Dvl and induces Dvl degradation via a lysosome inhibitor-sensitive and proteasome inhibitor-insensitive mechanism [42]. Dvl is a central mediator of Wnt signaling. It is activated by the binding of Wnt ligands to the Frizzled/low-density lipoprotein receptor-related protein (LRP) coreceptor at the cell surface. Activated Dvl binds to the axin/GSK-3β complex and inhibits the GSK-3β-induced degradation of β-catenin, therefore activating the expression of target genes via the β-catenin-T-cell factor-lymphoid enhancer factor complex [4], [15]. Our data showed that *DACT1* could downregulate the expression of Dvl-2 (Figure S4), whereas the expression of Dvl-1 and Dvl-3 was not been affected by *DACT1* (Figure S5). The results suggest that *DACT1* upregulates β-catenin level in colon cancer cells through direct regulation of the multiprotein complex and β-catenin. It is not clear whether *DACT1* regulates the phosphorylation of Dvl-2.

### *DACT1* mediated the subcellular localization of β-catenin

β-catenin can be found in three cell compartments: the plasma membrane, the cytoplasm and the nucleus. The soluble cytoplasmic pool of β-catenin is highly unstable; when it is stabilized by the Wnt signal, this results in nuclear localization. Nuclear β-catenin is a cofactor for a wide variety of transcription factors that regulate the expression of many genes involved in cell growth. At the membrane, β-catenin plays a pivotal role in cell-cell adhesion, linking the cytoplasmic domain of E-cadherin to α-catenin. The deregulation of β-catenin or E-cadherin leads to the loss of cell-cell adhesion [43]. We found that increased levels of *DACT1* led to higher levels of membrane-associated β-catenin, but the E-cadherin expression pattern in colon cancer cells was unaltered by *DACT1* siRNA or the overexpression of *DACT1*. We also found *DACT1* enhanced anchorage independence, as well as the migratory and invasive potential of colon cancer cell lines. These results are consistent with the above theories. In addition, our data showed that the *DACT1* expression level and membrane-associated β-catenin expression level were positively correlated in colon cancer cell lines and primary colon cancers. These results are consistent with our previous report that showed β-catenin was upregulated and is associated with *DACT1* levels. This demonstrates that there is a trend toward poor survival in patients with tumors that have higher scores for *DACT1* expression in colon adenocarcinomas [35].

### Mechanisms by which *DACT1* mediates the subcellular localization of β-catenin

Some reports have shown that β-catenin can shuttle in a bidirectional manner between the nucleus and cytoplasm, or between the nucleus and the cell membrane [44], [45], [46]. This is necessary to enable the cell to respond quickly to fluctuations in extracellular stimuli that require it to switch between nuclear transactivation and membrane cell adhesion or cell migration activities [30]. Previous studies have shown that the inhibition of GSK-3β promoted the accumulation of β-catenin in the nucleus [29] and at the plasma membrane [30], [31], which our data confirmed. In our studies, increased level of phosphorylated GSK-3β at Ser9 were present in HCT116 cells that expressed control siRNA, which led to the higher expression of β-catenin in the nucleus, cytoplasm and at the plasma membrane. We further found that *DACT1* interacted with GSK-3β. This result supports the finding that *DACT1* regulates the subcellular location of β-catenin through inhibiting the activity of GSK-3β. Reports have shown that Dvl can inhibit the activity of GSK-3β [47], [48]. Our studies showed that *DACT1* could downregulate the expression of Dvl-2, whereas the expression of Dvl-1 and Dvl-3 was not been affected by *DACT1* (Figure S4 and S5). These data further show that β-catenin accumulates in the nucleus and at the plasma membrane as a result of the inhibition of GSK-3β by *DACT1*.

### The model for the mechanism by which *DACT1* promotes the oncogenesis of colon cancer cells

**Figure 10:**
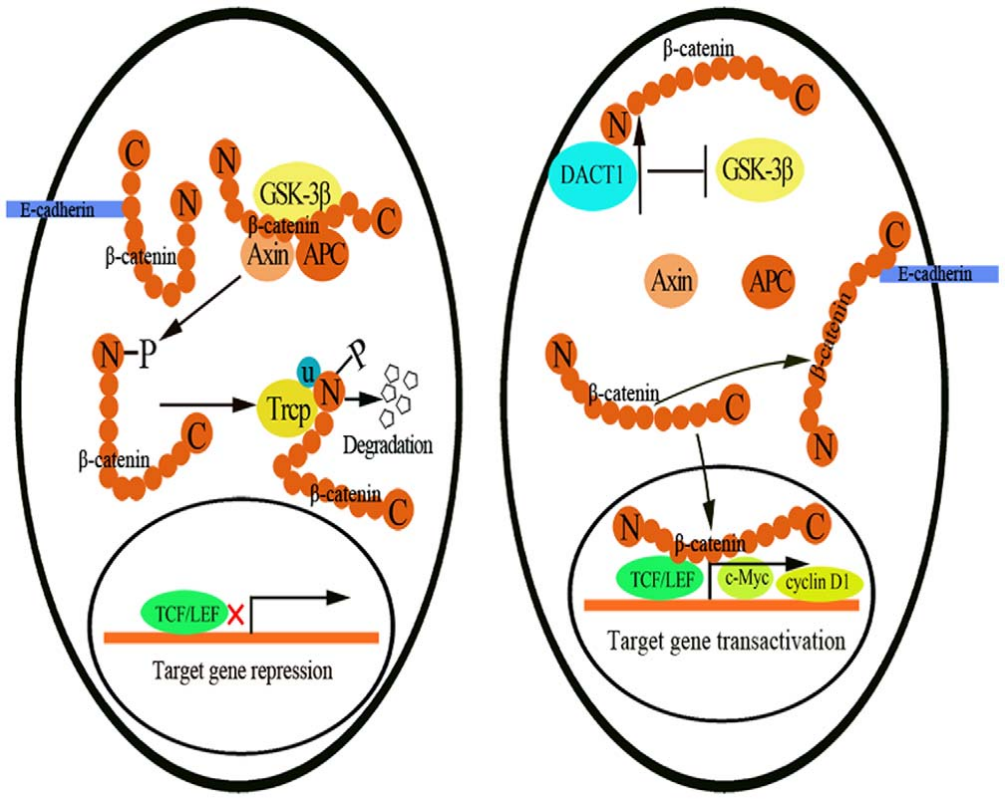
Putative model of how *DACT1* mediates the activation of β-catenin signaling in colon cancer cells. Left: In unstimulated normal colon cells lacking *DACT1*, the axin/GSK-3β/APC phosphorylate β-catenin and target it for ubiquitin-mediated degradation. Right: In colon cancer cells that express high levels of *DACT1*, *DACT1* binds to β-catenin. *DACT1* then binds and inhibits GSK-3β, which inhibits the function of the destruction complex, resulting in the release of β-catenin. This leads to increased nuclear and cytoplasmic fractions of β-catenin, and increased levels of membrane-bound β-catenin. Subsequently, the migration and invasion capacities of the colon cancer cells are enhanced and downstream target genes are activated.

The data in the present study support the following model for the mechanism by which *DACT1* promotes the oncogenesis of colon cancer cells (Figure 10). In unstimulated, normal colon cells that lack *DACT1*, GSK-3β is present in the cytoplasm and forms the β-catenin destruction complex with axin and APC. This allows the destruction complex to phosphorylate β-catenin and target it for ubiquitin-mediated degradation. In colon cancer cells that express high levels of *DACT1*, we propose that *DACT1* interacts with β-catenin, after which *DACT1* binds to and inhibits GSK-3β, which results in the release of β-catenin from the destruction complex, increased intracellular β-catenin levels, and its subsequent accumulation in the nucleus. This would lead to the activation of downstream β-catenin/TCF-regulated target genes, and at the membrane, regulation of the adhesion or migration properties of the cell. These studies define a functional role of *DACT1* in human tumorigenesis and, besides highlighting *DACT1* as a potential therapeutic target in colon cancer, define a mechanism for the regulation of β-catenin in cancer.

## Materials and Methods

### Tissue specimens and cell lines

Colon carcinoma tissues were obtained from the Peking University Third Hospital, China. Samples were frozen in liquid nitrogen immediately after surgical removal and maintained at 80°C until use. All human tissue was collected using protocols that were approved by the Ethics Committee of the Peking University Health Science Center. Patients diagnosed with colon adenocarcinoma were included if they had no family history of CRC or secondary malignancy, and had not received radiotherapy or chemotherapy before surgery. The human colon cancer cell lines were obtained from the cell resources centers of the Shanghai Institutes for Biological Sciences (Shanghai, China).

### Plasmid construction

The cDNA of human *DACT1* extracted from 293T cells was subcloned into the pEGFP-N1 expression vector (Invitrogen, Carlsbad, CA, USA). The sequences were verified by DNA sequencing. All of the control siRNAs and *DACT1* siRNAs constructs were kindly provided by Dr. Yeguang Chen (Tsinghua University, China).

### Quantitative real-time PCR and semi-reverse transcription-PCR

Total RNA from the human tissues and cell lines were isolated using TRIzol reagent (Invitrogen) according to the manufacturer's protocol. The cDNA of cell lines were synthesized using a High-Capacity cDNA Archive Kit (Invitrogen). Primer sequences for *DACT1* were as follows: sense, 5′- CACAAGCGAACTGACTACCG-3′; antisense, 5′-GTAATTGCTCTGCTCGTCCT-3′. The primers for GAPDH were: sense, 5′ –A CAG T C CATGCCATCACTGCC-3′; antisense, 5′-GCCTGCTTCACCACCTTCTTG-3′. Twenty-five cycles were used. The conditions for hot-start PCR were as follows: 95°C for 10 min, followed by amplification at 95°C for 1 min, 58°C for 30 s and 62°C for 30 s for DACT1 and GAPDH, respectively, and 72°C for 30 s, with a final extension at 72°C for 10 min.

The cDNA of human tissues served as a template in quantitative real-time PCR utilizing TaqMan Gene Expression Master Mix and TaqMan Gene Expression assay probes for *DACT1* (Hs00420410_m1), or *GAPDH* (Hs03929097_g1; Applied Biosystems, Foster City, CA, USA) and an ABI 7500 Fast Sequence Detection System. All reactions were performed in triplicate. The *DACT1* mRNA expression of different group specimens was normalized to endogenous GAPDH. The relative *DACT1* mRNA levels were presented as unit values of 2^−ΔCt^ = 2^−(Ct (GAPDH)−Ct (DACT1))^, where Ct is the threshold cycle value defined as the fractional cycle number at which the target fluorescent signal passes a fixed threshold above the baseline.

### Immunoblot analysis

After the frozen tissues were homogenized in liquid nitrogen and the cultured cells were digested by Trypsin/EDTA Solution (pH 7.4; 0.025% trypsin, 0.5 mM EDTA, 1 mM sodium pyruvate and 10 mM HEPES), whole-cell lysates were prepared by incubating cells in ice-cold lysis buffer (50 mM Tris-HCl [pH 8.0], 150 mM NaCl, 1% Triton X-100 and 100 µg/ml of phenylmethylsulfonyl fluoride) for 30 min. The tissue samples and cells were sonicated for 8 s and then placed on ice for 5 min. The lysates were then centrifuged at 12,000 g for 20 min at 4°C. The supernatant was collected, aliquotted and frozen at −80°C before use. Protein concentrations were determined with a protein assay kit (Bio-Rad Laboratories, Hercules, CA, USA) according to the protocol of the manufacturer. Equal amounts of protein were resolved by 10% sodium dodecyl sulfate-polyacrylamide gel electrophoresis (SDS-PAGE) and transferred to PVDF membranes (Millipore, Bedford, MA, USA). The antibodies used in these experiments included *DACT1* (1∶8,000; Abcam, Cambridge, UK), β-catenin (1∶4,000; Cell Signaling Technology, Beverly, MA, USA), c-Myc, cyclin D1, CDK4, GSK-3β, Axin1, (all diluted to 1∶2,000; Cell Signaling Technology), active-β-Catenin (1∶2,000; Millipore). After analysis, the blots were stripped, washed and re-probed with an antibody against GAPDH (Sigma, St. Louis, MO, USA), which served as the loading control. Images were visualized with an enhanced chemiluminescence (ECL) detection system (Amersham Biosciences UK, Ltd., Little Chalfont, Buckinghamshire, UK).

### Cell culture

All of the cells were cultured in Dulbecco's modified Eagle's medium supplemented with 10% fetal bovine serum (Invitrogen), nonessential amino acids, L-glutamine, and penicillin/streptomycin in an atmosphere that contained 5% CO_2_ at 37°C.

### Creation of stable cell lines

SW480 cells were stably transfected with pEGFP-N1-*DACT1* and pEGFP-N1 empty vectors so that they would stably overexpress *DACT1*. The selection for G418 resistance was initiated 72 h after transfection by the addition of 300 µg/ml of G418 (Invitrogen) to the culture medium. The selection media was changed every 3–4 days for several weeks, and clones of resistant G418 cells were isolated and expanded for further characterization. *DACT1* expression in stably transfected cells was verified by Western blotting, as described above.

To silence *DACT1* expression, HCT116 and LoVo and HT29 cells were stably transfected with *DACT1* siRNA and control siRNA expression vectors. Selection for puromycin resistance was initiated 72 h after transfection by the addition of 5 µg/ml puromycin (Invitrogen) to the culture medium. The selection media was changed every 3–4 days for several weeks, and clones of puromycin-resistant cells were isolated and expanded for further characterization. The silencing of *DACT1* expression in stably transfected cells was verified by Western blotting, as described above.

### Proliferation assay

Cells were seeded in 24-well plates at 1×10^4^ cells per well in a volume of 1 ml of supplemented medium. The supplemented medium was changed every day. Cell numbers were counted every 24 h with the use of a NucleoCounter cell counter (Chemomete, Denmark).

### Soft agar colony assay

Cell suspensions (1,000 cells) were prepared using 0.6% Noble agarose (Becton Dickinson) and overlaid onto a 24-well plate that contained a solidified lower layer of 0.6% agarose in medium. Once the top layer had solidified, 1 ml of medium was placed on the top of the cell layer. After treatment, plates were incubated for 3 weeks and colonies were counted with the assistance of a stereomicroscope.

### Transwell migration assay

For transwell migration assays, 5×10^4^ cells were plated into the top chamber on a noncoated membrane (24-well insert; pore size, 8 µm; Corning Costar, NY, USA); cells were then allowed to migrate toward the medium that contained serum in the lower chamber. Cells were fixed with methanol after 24 h of incubation and stained with 0.1% crystal violet (2 mg/mL; Sigma-Aldrich). The number of cells that invaded through the membrane was counted under a light microscope (20×, three random fields per well).

### Transwell invasion assay

For the invasion assay, 5×10^4^ cells were plated in the top chamber onto the Matrigel-coated membrane (24-well insert; pore size, 8 µm; Millipore). Cells were plated in medium without serum, and medium supplemented with serum was used as a chemoattractant in the lower chamber. The cells were incubated for 24 h and cells that did invade through the pores were removed with a cotton swab. Cells on the lower surface of the membrane were fixed with methanol and stained with crystal violet. The number of cells invading through the membrane was counted under light microscopy (20×, three random fields per well).

### Flow cytometry analysis

After all of the cells were synchronized to the G0/G1 phases by serum starvation, the cells were cultured for 72 h. Then, the cells were washed twice with ice-cold phosphate buffered saline (PBS), harvested, fixed with ice-cold PBS in 70% ethanol, and stored at −20°C for 30 min. After fixation, the cells were incubated with RNase A (0.1 mg/ml, Sigma-Aldrich) at 37°C for 30 min, stained with propidium iodide (50 µg/ml, Sigma-Aldrich) for 30 min on ice in the dark and analyzed for DNA content using a flow cytometer (FACSCalibur; BD Biosciences). Data were collected in list mode for at least on 10,000 events and analyzed using Mod Fit 2.0 software.

### Immunohistochemical analysis

The paraffin-embedded colon tissue sections (4-µm thick) were cut, deparaffinized, and subjected to a heat-induced epitope retrieval step. Endogenous peroxidase activity was blocked with 1% (v/v) hydrogen peroxide in distilled water. To block nonspecific binding, the sections were incubated with 2% (g/v) BSA for 60 min at room temperature. Subsequently, samples were incubated with a rabbit polyclonal β-catenin antibody (Cell Signaling Technology), diluted to 1∶200 in PBS for spending the night at 4°C. For negative controls, sections were incubated with PBS without the primary antibody. For detection, specimens were sequentially incubated with biotinylated mouse anti-rabbit immunoglobulin G (IgG) and streptavidin-horseradish peroxidase. β-catenin expression levels in the stained sections were evaluated by an experienced pathologist.

### Immunofluorescence microscopy

The OCT (optimal cutting temperature)-embedded colon tissue sections (4-µm thick) were cut. Cells were grown on a confocal dish (1×10^5^/mL) and allowed to grow for 48 h. The sections and cells were then fixed for 10 min in ice-cold 4% paraformaldehyde with 0.2% Triton-100. The sections and cells were washed in PBS and incubated for 1 h in TBS containing 5% BSA. Sections and cells were incubated overnight at 4°C with a β-catenin antibody (1∶200 dilution), E-cadherin antibody (1∶200 dilution), or phospho-GSK-3β(Ser9) (1∶100; Cell Signaling Technology), active β-catenin antibody (1∶200 dilution; Millipore). For the double-immunolabelling experiments, the primary antibodies against GFP (anti-mouse; 1∶50), β-catenin antibody (anti-rabbit; 1∶200); axin1 antibody (anti-rabbit; 1∶200), GSK-3β (anti-rabbit; 1∶200) and *DACT1* antibody (anti-rabbit; 1∶200), β-catenin antibody (anti-mouse; 1∶200) or GSK-3β (anti-mouse; 1∶200; Santa Cruz) were incubated simultaneously overnight at 4°C. Sections and cells grown on the confocal dish were rinsed at least three times for 5 min in PBS, and were then simultaneously incubated with the secondary antibody fluorescein (FITC)-conjugated anti-mouse IgG (Jackson, West Grove, PA, USA), or Dyelight594-conjugated anti-rabbit IgG (Jackson) at a 1∶1,000 dilution for 1 h at room temperature. Sections and cells were then counterstained with 4′,6-diamidino-2-phenylindole (DAPI) and mounted with Vectashield (Vector Laboratories, Burlingame, CA). Immunofluorescence was visualized using a laser confocal scanning microscope (LSM 510 Meta).

### Nuclear and membrane fractionation

Nuclear and cytoplasmic proteins were extracted using a NE-PERN Nuclear and Cytoplasmic Extraction Reagents Kit (Pierce, Rockford, IL, USA). Membrane proteins were extracted using a Mem-PER Eukaryotic Membrane Protein Extraction Kit (Pierce), according to the instructions of the manufacturer.

### Immunoprecipitation

The cells were lysed with 1 ml of lysis buffer (20 mM Tris-HCl, pH 7.4, 2 mM EDTA, 25 mM NaF, 1% Triton X-100) plus protease inhibitors (Sigma) for 30 min at 4°C. After 12,000 g centrifugation for 15 min, the lysates were immunoprecipitated with specific antibody and protein A-Sepharose (Zymed Laboratories Inc., San Francisco, CA, USA) for 3 h at 4°C. Thereafter, the precipitants were washed three times with washing buffer (50 mM Tris-HCl, pH 8.0, 15 mM NaCl, 1% Nonidet P-40, 0.5% sodium deoxycholate, and 0.1% SDS). The immune complexes were then eluted with sample buffer containing 1% SDS for 5 min at 95°C and analyzed by SDS-PAGE.

### *In vivo* tumorigenicity studies

Six- to 8-week-old female nude mice were obtained from the Animal Center of Peking University Health Science Center (Beijing, China). Eight animals per group were used in each experiment. Briefly, 5×10^6^ HCT116 cells (expressing control siRNA or *DACT1* siRNA) in 200 µl PBS were injected into the subcutaneous tissue of the nude mouse. Tumors were measured weekly using a vernier caliper, and the tumor volume was calculated according to the following formula: π/6×length×width^2^ [21]. Tumors were collected at the time of sacrifice 8 weeks after infection. All studies were approved by the Animal Care Committee of Peking University Health Science Center.

### Statistical analysis

Data are represented as mean ± standard error of the mean (SEM) from at least three independent experiments. The significance of differences between groups was evaluated by Student's *t*-test or ANOVA. P-values<0.05 were considered to be significant.

## Supporting Information

Figure S1 **The effects of** ***DACT1*** **on cellular proliferation and β-catenin levels in HCT116 cells.** (A) DACT1 expression in wild-type, control siRNA-transfected, and *DACT1* siRNA1-transfected HCT116 cells. (B) Growth curve assays in *DACT1* siRNA1-transfected HCT116 cells (mean ± SEM; n = 3, *p<0.05 versus wild-type cells). (C) Soft agar assay in siRNA1-transfected HCT116 cells after 14 days of incubation. (D) Photomicrographs of control siRNA and *DACT1* siRNA1 in HCT116 cells immunostained with an anti-β-catenin antibody (red).
(TIF)

Figure S2 **Representative Western blots showing CDK4 expression levels in colon cancer cells.** “+” represents the overexpression of *DACT1* in SW480 cells and control siRNA in HCT116, LoVo and HT29 cells. “−” represents empty vector in SW480 cells and *DACT1* siRNA in HCT116, LoVo and HT29 cells.
(TIF)

Figure S3 **A representative photomicrograph immunostained with anti-E-cadherin antibody (green) in colon cancer cells.**
(TIF)

Figure S4 **A representative photomicrograph immunostained with anti-Dvl-2 antibody (red) in colon cancer cells.**
(TIF)

Figure S5 **Representative Western blots showing Dvl-1 and Dvl-3 expression levels in colon cancer cells.** “+” represents the overexpression *DACT1* in SW480 cells and control siRNA in HCT116, LoVo and HT29 cells. “−” represents empty vector in SW480 cells and *DACT1* siRNA in HCT116, LoVo and HT29 cells.
(TIF)
