# Supplementary figures and images for: Oncogenic Function of DACT1 in Colon Cancer through the Regulation of β-catenin
Source: PLoS One. 2012 Mar 21;7(3):e34004. doi: 10.1371/journal.pone.0034004 (PMC3309901; doi:10.1371/journal.pone.0034004)

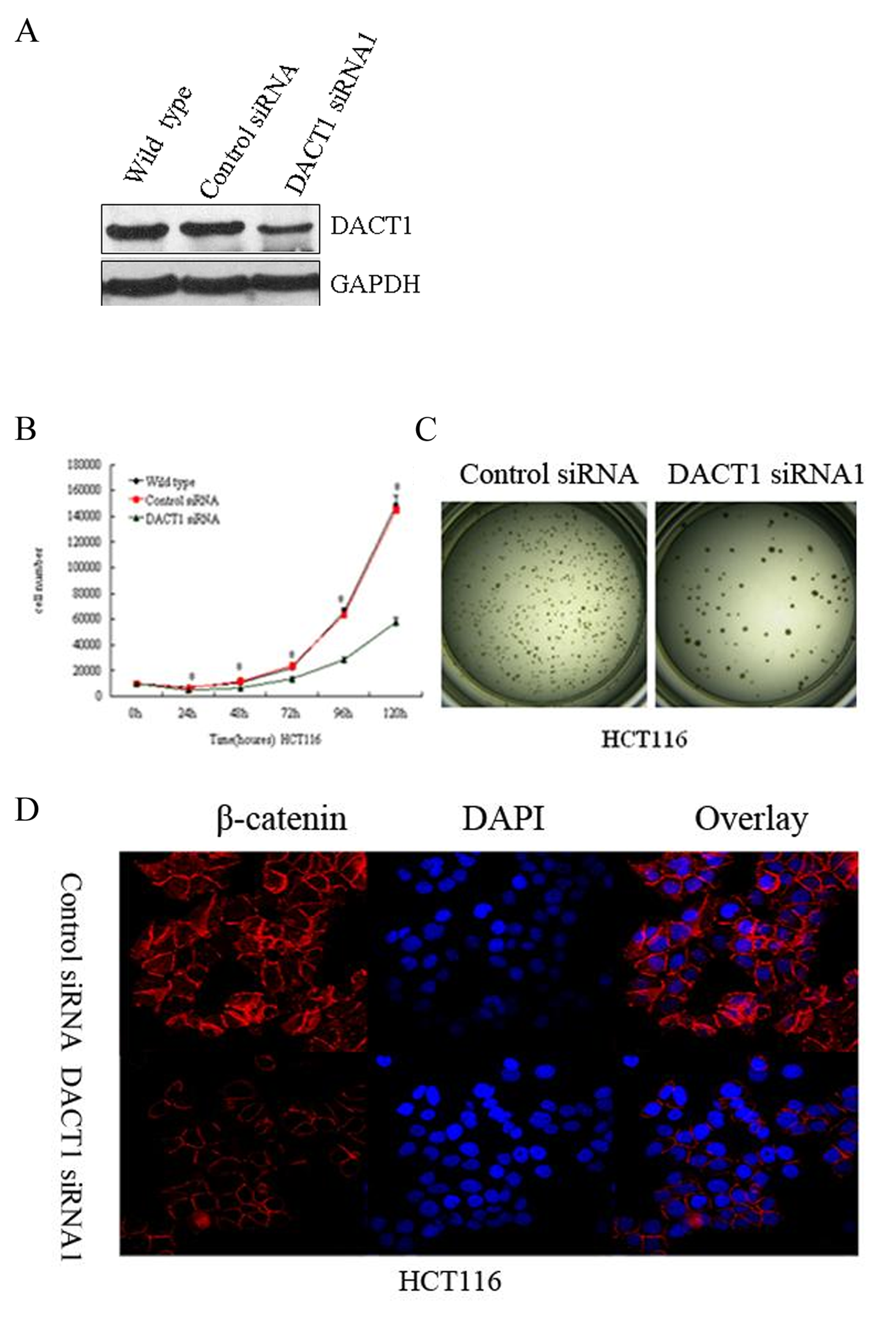

Supplement: Figure S1 — The effects of DACT1 on cellular proliferation and β-catenin levels in HCT116 cells. (A) DACT1 expression in wild-type, control siRNA-transfected, and DACT1 siRNA1-transfected HCT116 cells. (B) Growth curve assays in DACT1 siRNA1-transfected HCT116 cells (mean ± SEM; n = 3, *p<0.05 versus wild-type cells). (C) Soft agar assay in siRNA1-transfected HCT116 cells after 14 days of incubation. (D) Photomicrographs of control siRNA and DACT1 siRNA1 in HCT116 cells immunostained with an anti-β-catenin antibody (red). (TIF) [file pone.0034004.s001.tif]

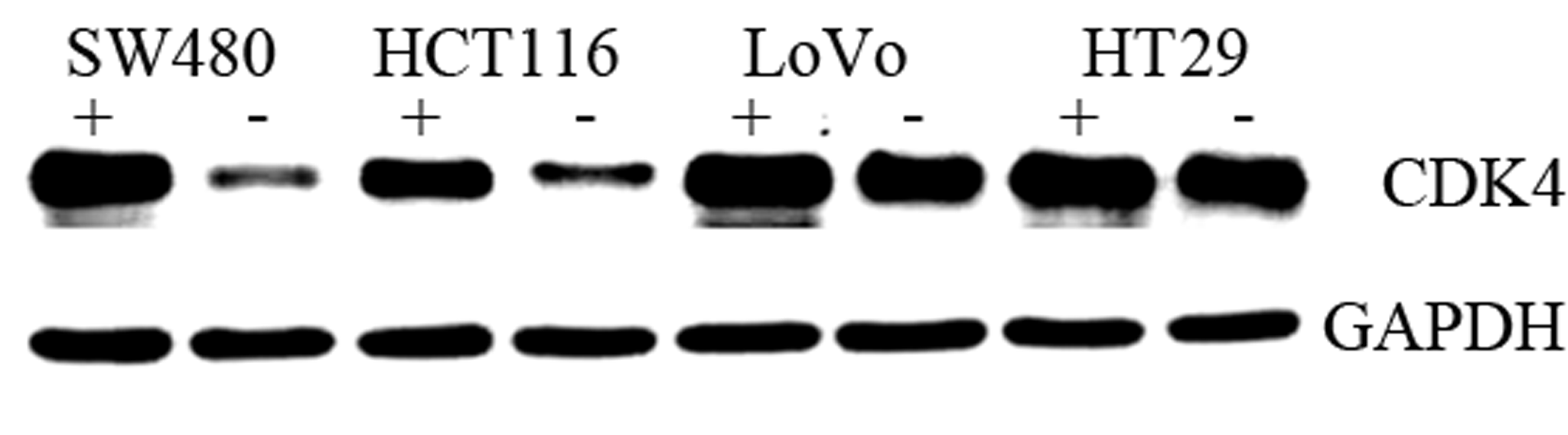

Supplement: Figure S2 — Representative Western blots showing CDK4 expression levels in colon cancer cells. “+” represents the overexpression of DACT1 in SW480 cells and control siRNA in HCT116, LoVo and HT29 cells. “−” represents empty vector in SW480 cells and DACT1 siRNA in HCT116, LoVo and HT29 cells. (TIF) [file pone.0034004.s002.tif]

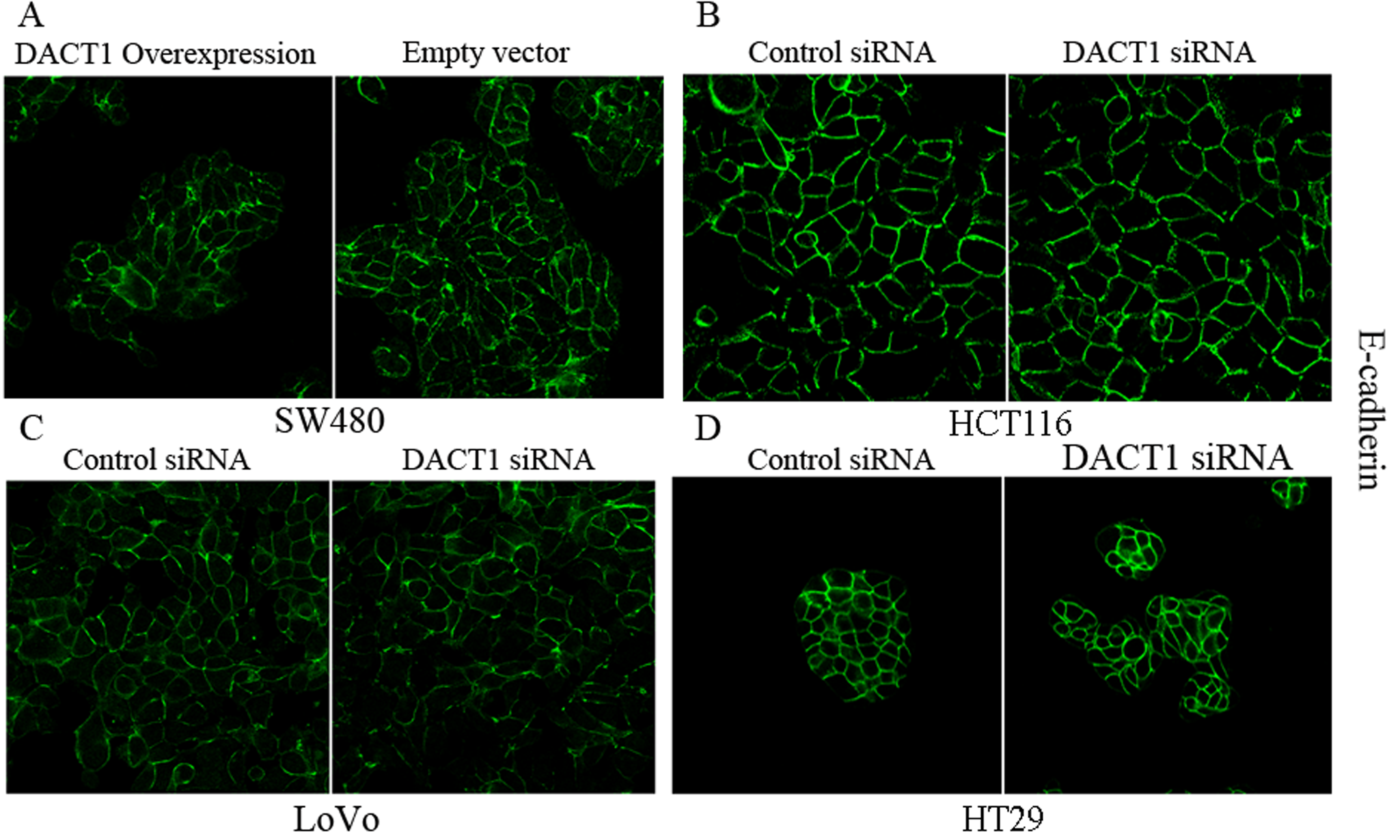

Supplement: Figure S3 — A representative photomicrograph immunostained with anti-E-cadherin antibody (green) in colon cancer cells. (TIF) [file pone.0034004.s003.tif]

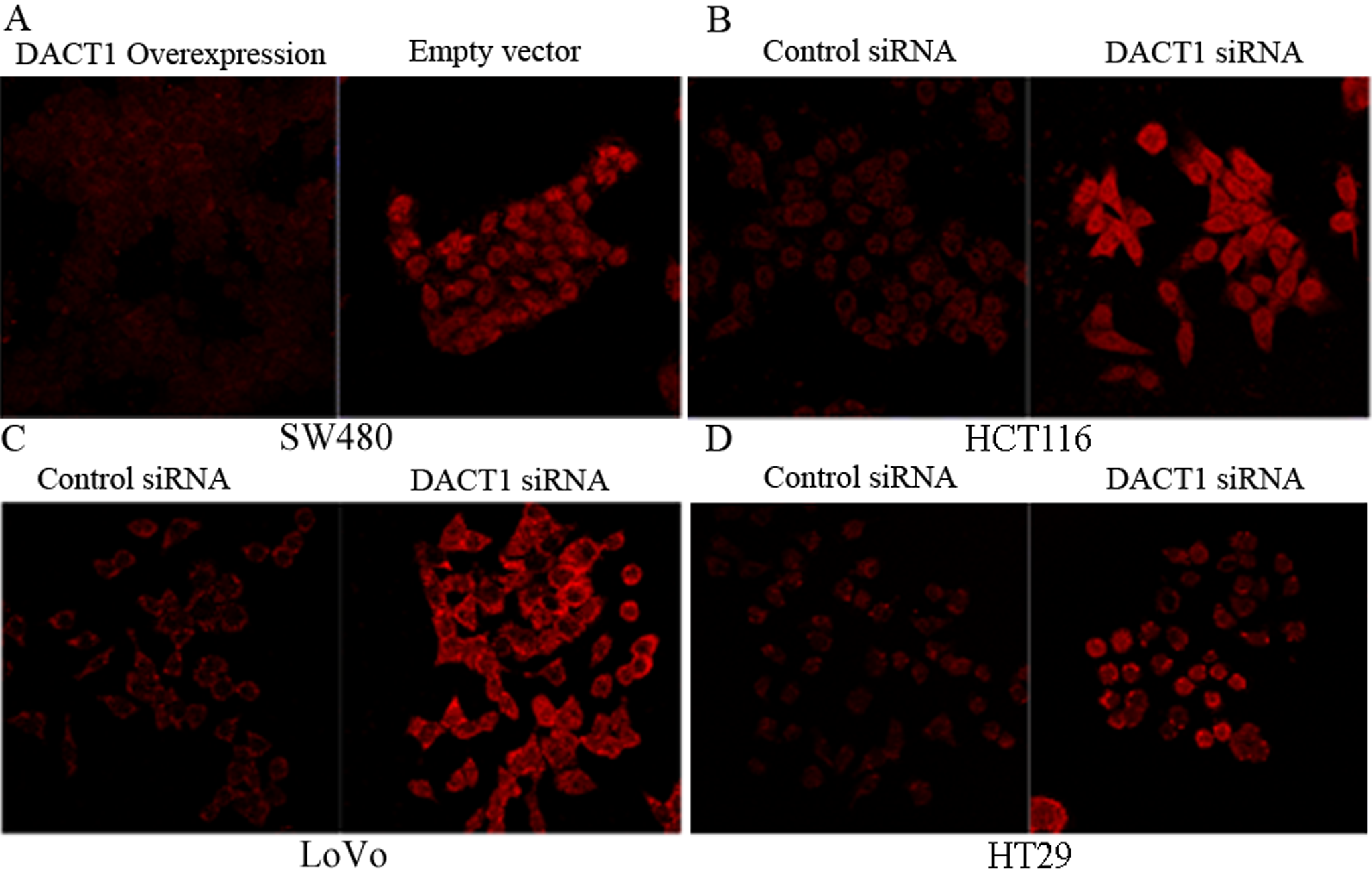

Supplement: Figure S4 — A representative photomicrograph immunostained with anti-Dvl-2 antibody (red) in colon cancer cells. (TIF) [file pone.0034004.s004.tif]

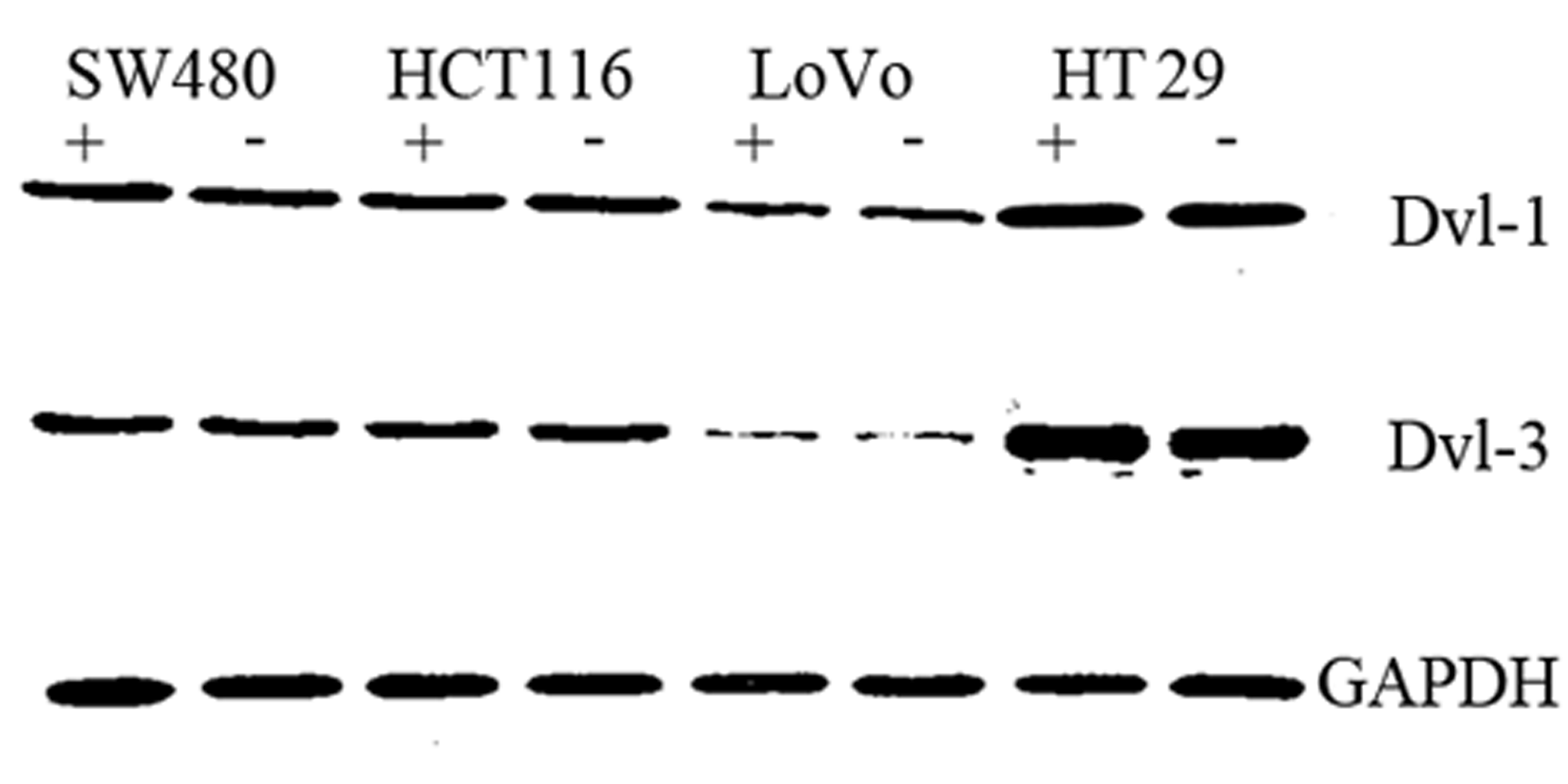

Supplement: Figure S5 — Representative Western blots showing Dvl-1 and Dvl-3 expression levels in colon cancer cells. “+” represents the overexpression DACT1 in SW480 cells and control siRNA in HCT116, LoVo and HT29 cells. “−” represents empty vector in SW480 cells and DACT1 siRNA in HCT116, LoVo and HT29 cells. (TIF) [file pone.0034004.s005.tif]
